# Behavioral Dynamics of AI Trust and Health Care Delays Among Adults: Integrated Cross-Sectional Survey and Agent-Based Modeling Study

**DOI:** 10.2196/82170

**Published:** 2026-02-03

**Authors:** Xueyao Cai, Weidong Li, Wenjun Shi, Yuchen Cai, Jianda Zhou

**Affiliations:** 1 Department of Plastic Surgery The Third Xiangya Hospital Central South University Changsha China; 2 Department of Plastic and Reconstructive Surgery Shanghai Ninth People’s Hospital Shanghai Jiao Tong University School of Medicine Shanghai China

**Keywords:** agent-based modeling, artificial intelligence, chronic disease, health care delay, real-world research

## Abstract

**Background:**

While artificial intelligence (AI) holds significant promise for health care, excessive trust in these tools may unintentionally delay patients from seeking professional care, particularly among patients with chronic illnesses. However, the behavioral dynamics underlying this phenomenon remain poorly understood.

**Objective:**

This study aims to quantify the influence of AI trust on health care delays through integrated survey-based mediation analysis and real-world research, and to simulate intervention efficacy using agent-based modeling (ABM).

**Methods:**

A cross-sectional online survey was conducted in China from December 2024 to May 2025. Participants were recruited via convenience sampling on social media (WeChat and QQ) and hospital portals. The survey included a 21-item questionnaire measuring AI trust (5-point Likert scale), AI usage frequency (6-point scale), chronic disease status (physician-diagnosed, binary), and self-reported health care delay (binary). Responses with completion time <90 seconds, logical inconsistencies, missing values, or duplicates were excluded. Analyses included descriptive statistics, multivariable logistic regression (α=.05), mediation analysis with nonparametric bootstrapping (500 iterations), and moderation testing. Subsequently, an ABM simulated 2460 agents within a small-world network over 14 days to model behavioral feedback and test 3 interventions: broadcast messaging, behavioral reward, and network rewiring.

**Results:**

The final sample included 2460 adults (mean age 34.46, SD 11.62 years; n=1345, 54.7% female). Higher AI trust was associated with increased odds of delays (odds ratio [OR] 1.09, 95% CI 1.00-1.18; *P*=.04), with usage frequency partially mediating this relationship (indirect OR 1.24, 95% CI 1.20-1.29; *P*<.001). Chronic disease status amplified the delay odds (OR 1.42, 95% CI 1.09-1.86; *P*=.01). The ABM demonstrated a bidirectional trust erosion loop, with population delay rates declining from 10.6% to 9.5% as mean AI trust decreased from 1.91 to 1.52. Interventions simulation found broadcast messaging most effective in reducing delay odds (OR 0.94, 95% CI 0.94-0.95; *P*<.001), whereas network rewiring increased odds (OR 1.04, 95% CI 1.04-1.05; *P*<.001), suggesting a “trust polarization” effect.

**Conclusions:**

This study reveals a nuanced relationship between AI trust and delayed health care–seeking. While trust in AI enhances engagement, it can also lead to delayed care, particularly among patients with chronic conditions or frequent AI users. Integrating survey data with ABM highlights how AI trust and delay behaviors can strengthen one another over time. Our findings indicate that AI health tools should prioritize calibrated decision support rather than full automation to balance autonomy, odds, and decision quality in digital health. Unlike previous studies that focus solely on static associations, this research emphasizes the dynamic interactions between AI trust and delay behaviors.

## Introduction

### Background

With the rapid development and widespread adoption of artificial intelligence (AI) in the medical field, AI has emerged as a pivotal tool across multiple domains, including health management, disease prediction, and clinical decision support [1,2]. Its potential to enhance health care efficiency, assist in disease diagnosis, optimize treatment strategies, and facilitate personalized health management is substantial [3,4]. However, this promise is tempered by growing concerns about its psychosocial and behavioral effects. The prevailing view of AI as an unequivocal force for good is being challenged by evidence that human-AI interaction can lead to new cognitive and behavioral issues [5,6]. In particular, AI’s capacity to provide health recommendations and interventions based on extensive data analysis marks a significant breakthrough [7,8]. However, this ability may alter traditional health decision-making processes. Trust is a key concept for adopting technology, involving aspects such as performance, process, and purpose. A growing body of research indicates that while AI offers convenient access to health-related information, excessive reliance on these tools may lead to detrimental health behaviors, such as overreliance on automated suggestions and disregarding clinical intuition [9-11].

Traditional health decision-making models typically depend on professional medical judgment and individuals’ self-awareness of their health status [12]. The advent of AI is reshaping this framework by providing automated diagnostic recommendations and personalized health guidance [13]. While such “digital health advice” can enhance confidence in medical decision-making in the short term, it may foster excessive dependence on AI tools over the long term. Patients with chronic diseases, who require ongoing health management and exhibit relatively stable symptoms, may be particularly susceptible to this shift [14]. This population often practices prolonged self-management, leading to frequent use of digital tools [15]. The relative stability of their conditions can create a sense of security, in which they may consider AI-generated reassuring feedback adequate. This dependence on AI could lead to delays in pursuing health care, as patients with chronic disease tend to give priority to AI-generated symptom evaluations and treatment options rather than traditional medical advice [16]. Consequently, they might overlook complex diagnostic and therapeutic needs that AI cannot fully assess [17]. Moreover, biases in medical AI can persist throughout its life cycle, potentially leading to serious repercussions in clinical decision-making. If these biases are not addressed, they can result in inaccurate medical judgments and exacerbate existing health care disparities [18]. While existing research has shed light on the potential risks of AI reliance in health care, a comprehensive framework that formally analyzes the complex relationship between AI trust and health behavior, particularly in the context of chronic disease, is still lacking. Additionally, few studies have used computational simulation to assess how various public health intervention strategies might mitigate this system-level risk.

**Figure 1:**
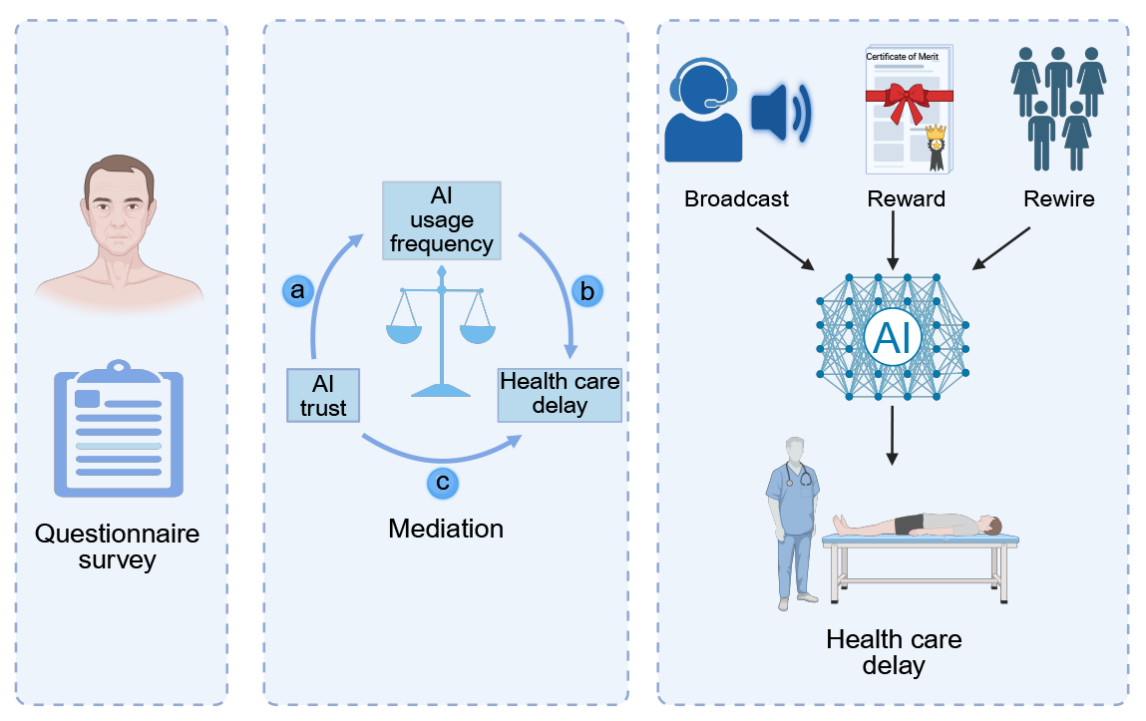
Schematic overview of the study design and methodology. Based on online questionnaires, the analysis framework includes mediation analysis to explore the relationship between artificial intelligence (AI) trust, usage frequency, and delayed health care–seeking behavior. Additionally, agent-based modeling was used to model the dynamic feedback loops over a 14-day period, incorporating 3 intervention strategies: broadcast, reward, and rewire. These strategies aimed to evaluate how the interventions affected trust and delay behavior within the population. Image created in Biorender by WL [19].

This study aims to systematically analyze the interrelationships among AI trust, AI usage frequency, chronic disease status, and delayed health care–seeking behavior. The analysis was conducted through a cross-sectional online survey from December 2024 to May 2025. We propose that AI trust and frequency of use jointly contribute to the odds of delayed medical care through a behavioral feedback mechanism, particularly pronounced among patients with chronic conditions. High levels of AI trust may inadvertently lead individuals to postpone health care–seeking by increasing usage frequency, highlighting a complex interaction between trust and behavior. To investigate these dynamic interdependencies, we use an agent-based modeling (ABM) framework to simulate the trust-behavior feedback over time. By embedding individuals within social networks and allowing trust and delay behaviors to evolve together, the ABM enables us to examine how microlevel decisions accumulate into population-level odds. This includes issues such as collective delays or systemic trust collapse, which are often difficult to capture using conventional cross-sectional data. A schematic overview of the study design is provided in Figure 1.

### Objective

This study not only addresses a theoretical gap in understanding the impact of AI on delayed health care–seeking but also offers a fresh perspective on optimizing the design of AI-based health tools. A key challenge in future medical applications of AI will be achieving the right balance between fostering user trust and ensuring accuracy and timeliness in health care–seeking. We aim to provide theoretical support for designing AI-driven health interventions. Additionally, we seek to inform more effective public health strategies, ensuring that technological advancements promote rather than hinder appropriate health care decisions.

## Methods

### Survey Design and Data Collection

This study was conducted in 2 integrated phases: a cross-sectional survey and an ABM simulation. To ensure comprehensive and transparent reporting, the methods are reported following the Journal Article Reporting Standards guidelines under relevant subheadings [20,21]. A cross-sectional online survey was conducted between December 1, 2024, and May 20, 2025. The 21-item questionnaire was conducted in Mandarin Chinese and developed based on a systematic literature review and focus-group discussions. It underwent refinement through 2 rounds of expert review by 4 specialists in public health and AI, and was finalized after a pilot test involving 5 target participants. The full survey questionnaire (English translation) is provided in Multimedia Appendix 1.

Eligible participants were adults aged 18-75 years who had resided in mainland China for the past 12 months and were able to read Chinese. The age range was selected to include the digitally engaged adult population while excluding minors and the very old people, who may exhibit different health-seeking patterns. The 12-month residency criterion ensured consistent exposure to the local health care, minimizing confounding from recent immigration or transient populations. The survey was disseminated via WeChat (Tencent) and QQ (Tencent), and the patient-education portals of collaborating hospitals using convenience sampling. Participants accessed the questionnaire after reading an electronic informed consent form and selecting “Agree.” All items were mandatory, and skip logic was applied to minimize irrelevant questions. Responses were transmitted over HTTPS and encrypted at rest on the institution’s private server, accessible only to authorized investigators.

To ensure the validity and interpretability of the logistic regression and mediation models, which require complete-case data, we performed rigorous data cleaning. Questionnaires completed in under 90 seconds, or those containing logical inconsistencies, missing values, or duplicate entries (only the first complete entry was retained), were excluded. Given the limited amount of missing data and the potential risk of introducing bias through imputation, listwise deletion was considered the most appropriate and conservative approach. To examine the mechanism of missingness, we conducted Little’s missing completely at random (MCAR) test using the *mice* package in R software (versions 4.2.3; R Foundation for Statistical Computing). Descriptive statistics were used to summarize the data (frequencies, percentages, means, and SDs), and multivariable logistic regression was conducted to examine the association between AI usage frequency and delayed health care–seeking behavior. Likert-type scales ranging from 1 to 5 (or 0 to 5 for AI usage and trust items) were used to assess frequency, trust level, and willingness to recommend.

Chronic disease was defined as any physician-diagnosed condition lasting 6 months or longer, including chronic allergic rhinitis, asthma, hypertension, diabetes, chronic skin diseases, etc. The variables included demographic characteristics (eg, age, sex, and occupation), AI usage behaviors (eg, frequency and exposure), trust perceptions, and health care–seeking outcomes.

Self-reported health care delay was assessed using a single binary item specifically targeting AI-influenced delay behaviors. Participants were asked: “Has advice provided by ChatGPT ever caused you to postpone or cancel seeking medical care?” with response options 0=no and 1=yes. This measure captures intentional delay or avoidance of health care that participants attributed directly to ChatGPT’s recommendations, rather than delays caused by logistical or accessibility barriers. This operationalization aligns with behavioral delay frameworks used in prior studies and reflects clinically meaningful patterns of health care–seeking behavior.

### Ethical Considerations

The study protocol was approved by the Institutional Review Board of Xiangya Third Hospital, Central South University (approval number 2025255). All procedures complied with the Declaration of Helsinki and the Personal Information Protection Law of China. Electronic informed consent was obtained from all participants. After reading a digital information sheet that outlined the study’s purpose, procedures, risks, benefits, and their rights (including voluntary participation and withdrawal), participants indicated their agreement by selecting “Agree” before proceeding to the questionnaire. All collected data were deidentified. No personally identifiable information (eg, name, ID number, and contact details) was stored with the response data. Data were transmitted securely via HTTPS and stored in encrypted form on a private, access-controlled institutional server. Results are reported in aggregate to prevent any possibility of individual identification. Participants did not receive any financial or material compensation for their involvement in this study. The manuscript and its supplementary materials do not contain any images, videos, or textual data that could lead to the identification of an individual participant. Therefore, specific consent for the publication of identifiable information was not applicable.

### Key Predictors of Delayed Care: Logistic Regression Analysis

We used logistic regression models to evaluate key predictors of delayed health care–seeking behavior. Initially, univariate logistic analyses were conducted to estimate the association between each candidate variable and the outcome, with results reported as odds ratios (ORs) accompanied by 95% CIs. Variables demonstrating potential significance were subsequently included in a series of hierarchical multivariate models (models 1-4). These models progressively incorporated individual characteristics, intervention exposure, AI usage patterns, and social influence to assess their independent contributions to delay behavior. This approach allowed us to identify the relative importance of each factor in influencing health care–seeking delays.

The data collection period encompassed the public release of DeepSeek, a major large language model in China, which occurred in early 2025 [22]. This event represented a significant shock to public awareness toward AI. To test the robustness of our core findings against this potential confounding effect, we performed a stratified analysis by dividing the sample into pre- and post-DeepSeek release subgroups. The cutoff date was set to February 1, 2025, allowing a sufficient time frame for the model’s public impact to materialize within our survey window. We then reran the univariate logistic analyses within each subgroup to assess the consistency of the associations between AI trust, usage frequency, and health care delays.

### Mediation Analysis: Indirect Effect of AI Trust via Usage Frequency

To examine whether AI trust influences delay behavior indirectly through AI usage frequency, we conducted mediation analysis using 2 approaches. First, following the traditional Baron and Kenny framework with the Sobel test, we estimated path *a* (the association between AI trust and usage frequency) via linear regression, and paths *b* and *c’* (the associations of usage frequency and AI trust with delay behavior) via multivariable logistic regression. The significance of the indirect effect (*a* × *b*) was assessed using the Sobel *z* test. Second, to obtain robust CIs and significance estimates, we performed nonparametric bootstrap resampling (n=500). In each iteration, we reestimated paths *a* and *b*, calculated the product *a* × *b*, and derived the empirical distribution of the indirect effect. The 95% bootstrap CI and *P* value were computed accordingly. All models were adjusted for age, gender, and chronic disease status.

### Moderation by AI Recommendation Exposure: Stratified and Interaction Models

To further examine whether the level of AI recommendation exposure moderates the relationship between AI trust and delayed health care–seeking behavior, we conducted stratified logistic regression and interaction modeling. Participants were divided into low and high exposure groups based on their reported frequency of receiving AI recommendations (≤2 vs >2). Separate multivariable logistic regression models were fitted for each group, adjusting for age, gender, chronic disease status, and AI usage frequency. ORs and 95% CIs were reported to assess effect heterogeneity across exposure levels.

Additionally, we introduced an interaction term between AI trust and recommendation exposure into the full model. The interaction coefficient was used to calculate the interaction OR and the corresponding 95% CI. Predicted probability curves were plotted to visualize how the relationship between AI trust and delay behavior varies by exposure group, providing insights into the moderating effect of AI recommendation exposure on health care–seeking behavior.

### Moderation by Recommendation Willingness: Stratified Trust-Delay Associations

To evaluate whether individuals’ willingness to recommend AI tools moderates the relationship between AI trust and delayed medical care, we conducted stratified logistic regressions and interaction analysis. Participants were grouped based on their scores (1-5) regarding the likelihood of recommending AI: those scoring ≤2 were classified as the “low recommendation intensity” group, and those scoring ≥3 as the “high recommendation intensity” group. Multivariable logistic regression models were fitted within each stratum, adjusting for age, gender, chronic disease status, and AI usage frequency. ORs and 95% CIs were calculated for AI trust in each group.

Subsequently, a full model including an interaction term between AI trust and recommendation intensity was constructed. We estimated the interaction effect (OR, 95% CI, and *P* value) and generated a prediction grid with standardized covariates to visualize the predicted probability of delayed care across AI trust levels in each group. Interaction plots were created to illustrate potential effect modification, enhancing our understanding of how willingness to recommend AI tools influences the relationship between AI trust and health care–seeking behavior.

### Scenario-Based Modeling: Joint Effects of AI Behavior Factors on Delay

To theoretically evaluate the potential impact of various intervention strategies on delayed health care–seeking behavior, we conducted a scenario-based simulation analysis using logistic regression. A multivariable logistic model was initially constructed with AI trust, frequency of use, and chronic disease status as predictors. ORs, 95% CIs, and *P* values were reported for each explanatory variable.

Based on this model, the following six intervention scenarios were simulated: (1) baseline (trust=3, frequency=3, no chronic disease); (2) increased AI trust (set to 5); (3) increased frequency of AI use (set to 5); (4) chronic disease status switched to “yes”; (5) combined trust + frequency increase; and (6) combined trust + frequency + chronic disease. For each scenario, predicted probabilities of delay were computed across all individuals and averaged to reflect group-level effects.

The results were visualized using a bar plot displaying the mean predicted probability of delay under each strategy. Annotations highlighted the ORs and significance levels of the 3 key predictors, facilitating an intuitive understanding of their relative contributions to delay behavior. This comprehensive approach allowed us to identify the most effective interventions for reducing delays in health care–seeking.

### ABM: Broadcast, Reward, Rewire

To evaluate the impact of different intervention mechanisms on delayed health care–seeking behavior, we used an ABM framework. ABM is a computational simulation approach particularly suited for studying complex systems, where population-level outcomes emerge from the interactions of autonomous, diverse individuals (“agents”) operating within a defined environment and set of rules [23-25]. This method is particularly appropriate for our research question for 3 main reasons. First, AI trust is not a static trait; it is a dynamic belief influenced by personal experience and social factors, which ABM is designed to capture. Second, the decision to delay care involves weighing personal trust against the behaviors of peers, a scenario well modeled by embedding agents within a social network where attitudes and actions spread [26,27]. Third, ABM allows us to test the “trust-delay” feedback loop, a causal chain that cannot be directly identified from our cross-sectional survey data but can be explored through simulation [28].

To improve the transparency and reproducibility, the ABM model is described following the overview, design concepts, and details (ODD) protocol [29]. The purpose of the simulation was to examine how AI trust, peer influence, and intervention strategies jointly shape delayed health care–seeking behavior over time. Each agent represented 1 survey respondent and was initialized using the individual’s empirical AI trust (1-5), AI usage frequency (1-5), and chronic disease status. Agents were embedded in a Watts-Strogatz small-world social network (n=2460; average degree=4; rewiring probability=0.2), which captures realistic social clustering and intermittent long-distance ties. The simulation progressed in daily cycles for 14 days, during which agents first computed a probability of delay using a logistic model derived from the survey data, then made a probabilistic delay decision, and subsequently updated their trust and usage behaviors according to personal outcomes, peer context, and intervention conditions. A total of 100 repetitions were performed for each scenario.

The model incorporated key design concepts of agent-based systems. Interaction occurred exclusively through local network neighbors; both average neighbor trust and neighbor delay behaviors influenced an individual’s own updates. Stochasticity was present in network initialization, delay decisions, and trial-level replications. Agents adapted their trust and usage frequency over time, increasing them when surrounded by high-trust peers or after not delaying care, and decreasing them when neighbors frequently delayed or after personally delaying care. No explicit learning mechanism was included; behavioral dynamics emerged entirely from rule-based adaptation. Model outputs consisted of daily population-level delay rates and comparative effects of intervention strategies relative to baseline.

Detailed implementation followed ODD guidelines [29]. At initialization, agents with an empirically predicted baseline delay probability above 0.20 were assigned a 1-point reduction in trust and usage frequency to represent structural vulnerability. During each daily update, delaying care reduced trust by 0.2 and usage frequency by 0.3, whereas not delaying increased trust by 0.1; all values were bounded between 1 and 5. A total of 3 intervention strategies were embedded into this baseline framework. In the broadcast condition, trust was reduced daily by a small fixed penalty to reflect exposure to trust-eroding messages. The reward condition increased trust and usage frequency for agents who consistently sought timely care, with rewards provided every 2 days. In the rewiring condition, network edges were periodically redirected toward the highest-trust agents to model opinion leader amplification. All intervention rules were implemented on top of the core behavioral update mechanism. Finally, 1-way sensitivity analyses were conducted by varying initial trust levels (means of 2.5, 3.0, and 3.5), broadcast penalty intensities (0.05-0.20 per day), reward magnitudes (0.03-0.10), and rewiring frequencies (intervals of 2-10 days). Each parameter set was simulated in 100 trials, and the resulting delay trajectories were compared to assess model robustness.

## Results

### Demographic Characteristics, AI Use, and Health Decision Outcomes

Of 2785 initial submissions, 325 (11.7%) responses were excluded based on prespecified criteria (completion time <90 seconds, logical inconsistencies, duplicate entries, or missing values in key variables). Specifically, 136 (4.9%) exclusions from the initial sample were due to missing values. Little’s MCAR test indicated that the data were MCAR (*χ*^2^_14_=12.87; *P*=.54), supporting the use of complete-case analysis. Consequently, a total of 2460 valid responses were retained, predominantly female (n=1345, 54.7%), with an average age of 34.46 (SD 11.62) years. Occupations included students (n=825, 33.5%), technology workers (n=715, 29.1%), other professions (n=640, 26%), and health care personnel (n=280, 11.4%). A significant majority (n=2215, 90%) reported being aware of generative AI tools, and 62% (n=1525) had previously used AI for health advice. Regarding the frequency of AI-based advice use, 38% (n=935) of participants never used it, while 16.2% (n=398) used it weekly (1-2 times) and 15.7% (n=385) monthly. Trust in AI-generated advice varied, with 38% (n=935) never using it, and the remaining participants distributed across differing trust levels (Table 1).

In terms of health care decision outcomes, 11.6% (285/2460) of participants deferred or canceled health care due to AI advice, while 18.9% (465/2460) changed their health care decisions based on it. Additionally, 16.9% (415/2460) adopted alternative therapies following AI recommendations. The primary source of health information was physicians (1203/2460, 48.9%), followed by search engines (465/2460, 18.9%) and generative AI tools (375/2460, 15.2%). Perceived influence of online discussions varied, with 19.7% (485/2460) considering it very likely to impact their decisions. Lastly, 30.1% (740/2460) of participants reported having a chronic disease (Table 2).

**Table 1:** Demographic characteristics and artificial intelligence (AI) use (n=2460).

Variable		Values
**Sex, n (%)**		
	Female	1345 (54.7)
	Male	1115 (45.3)
Age (years), mean (SD)		34.46 (1.62)
**Occupation category, n (%)**		
	Students	825 (33.5)
	Technology workers	715 (29.1)
	Other	640 (26)
	Health care personnel	280 (11.4)
**Awareness of generative-AI tools, n (%)**		
	Yes	2215 (90)
	No	245 (10)
**Previous use of AI for health advice, n (%)**		
	Yes	1525 (62)
	No	935 (38)
**Frequency of AI-based advice use, n (%)**		
	Never	935 (38)
	Weekly 1-2 times	398 (16.2)
	Monthly	385 (15.7)
	Weekly 3-4 times	308 (12.5)
	Occasional	293 (11.9)
	Almost daily	141 (5.7)
**Trust in AI-generated advice, n (%)**		
	1 (“none”)	320 (13)
	2	290 (11.8)
	3	280 (11.4)
	4	345 (14)
	5 (“high”)	290 (11.8)
	0 (“never used”)	935 (38)

**Table 2:** Health decision outcomes and contextual factors (n=2460).

Variable		Value, n (%)
**Deferred or cancelled health care due to AI^a^ advice**		
	Yes	285 (11.6)
	No	2175 (88.4)
**Changed health care decision due to AI advice**		
	Yes	465 (18.9)
	No	1995 (81.1)
**Adopted alternative therapy due to AI advice**		
	Yes	415 (16.9)
	No	2045 (83.1)
**Primary source of health information**		
	Physician	1203 (48.9)
	Search engines	465 (18.9)
	Generative AI tools	375 (15.2)
	Family or friends	255 (10.3)
	Government or traditional media	85 (3.5)
	Social networking	77 (3.1)
**Perceived influence of online discussions**		
	Very likely	485 (19.7)
	Likely	835 (33.9)
	Uncertain	775 (31.5)
	Unlikely	265 (10.8)
	Very unlikely	100 (4.1)
**Presence of chronic disease**		
	Yes	740 (30.1)
	No	1720 (69.9)

^a^AI: artificial intelligence.

### AI Trust and Usage Frequency Are Key Predictors of Delay

**Figure 2:**
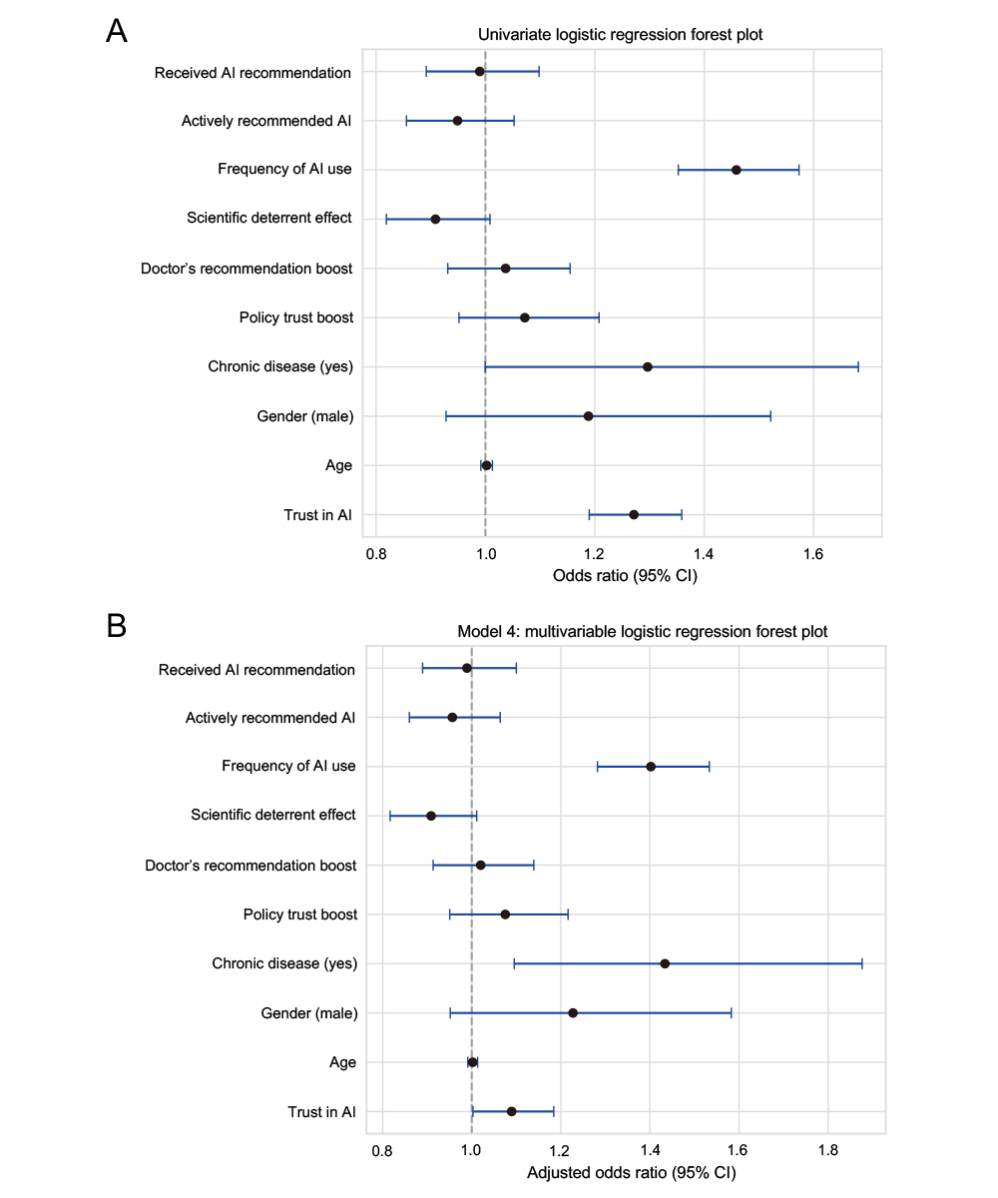
Association between artificial intelligence (AI) trust, usage frequency, and delayed health care–seeking behavior. (A) Univariate logistic regression and (B) fully adjusted multivariable model evaluating key predictors of delayed health care–seeking behavior.

In the univariate logistic regression, higher AI trust was positively associated with delayed health care–seeking behavior, with an OR of 1.27 (95% CI 1.19-1.36; *P*<.001; Figure 2A). Similarly, the frequency of AI use also showed a significant positive correlation with delay (OR 1.41, 95% CI 1.18-1.69; *P*<.001; Figure 2A). Although not statistically significant, trends were observed in the effects of receiving AI recommendations (OR 0.95, 95% CI 0.81-1.11) and actively recommending AI (OR 0.92, 95% CI 0.781.09; Figure 2A), both suggesting a potential reduction in delay. In the fully adjusted model (model 4), AI trust remained a significant predictor (OR 1.09, 95% CI 1.00-1.18; *P*=.04; Figure 2B). Frequency of AI use exhibited the strongest association with delay (OR 1.39, 95% CI 1.14-1.68; *P*<.001; Figure 2B), indicating that more frequent users had higher odds of postponing medical visits. Additionally, chronic disease status demonstrated a persistent positive association with delay (OR 1.43, 95% CI 1.10-1.88; *P*=.009; Figure 2B). Detailed analyses for univariate and multivariate logistic regression can be found in Tables S1 and S2 in Multimedia Appendix 2.

To address potential confounding from a major AI market event, we stratified the analysis by the DeepSeek release period. The results confirmed the robustness of our primary findings. The association between AI usage frequency and delayed health care–seeking remained nearly identical in both direction and magnitude in the pre- and postrelease periods (prerelease: OR 1.41, 95% CI 1.17-1.70; *P*<.001; postrelease: OR 1.41, 95% CI 1.27-1.56; *P*<.001). For AI trust, while the positive association was slightly attenuated and not statistically significant in the prerelease period (OR 1.05, 95% CI 0.89-1.25; *P*=.56), it was significant in the postrelease period (OR 1.10, 95% CI 1.00-1.21; *P*=.046). This suggests that the fundamental behavioral mechanism linking AI trust and usage to delay is robust. The DeepSeek release may have slightly amplified the measurable effect of AI trust, possibly due to increased public reliance on AI tools. Detailed results of this stratified analysis are available in Table S3 in Multimedia Appendix 2.

### AI Trust Influences Delay via Frequency: Evidence of Mediation

**Figure 3:**
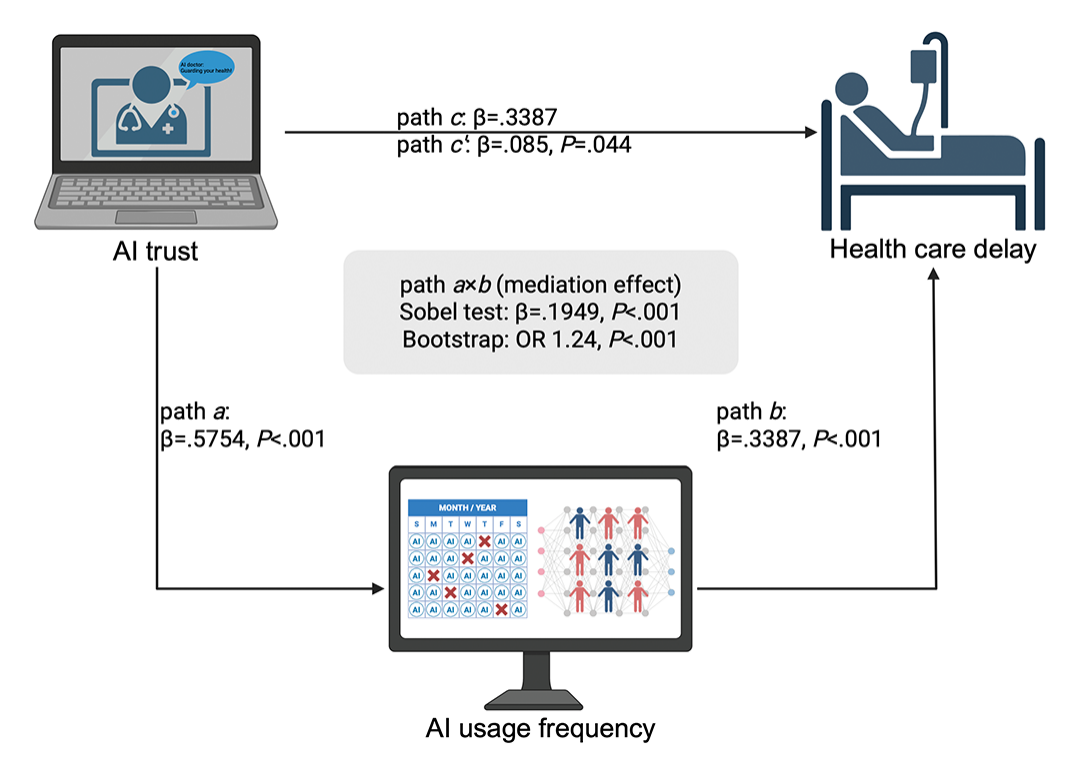
Mediation model demonstrating how artificial intelligence (AI) trust influences delayed health care–seeking through increased frequency of AI usage. Path *a* shows the significant effect of AI trust on usage frequency, while path *b* indicates the association between usage frequency and delay. The indirect effect (*a* × *b*) and direct effect (*c’*) are also represented, highlighting the statistical significance of the mediation pathway. Image created in Biorender by WL [30]. OR: odds ratio.

We further evaluated the indirect effect of AI trust on delayed health care–seeking through AI usage frequency, supporting a partial mediation model. In the linear regression analysis, AI trust significantly predicted usage frequency (path *a*: β=.5754; *P*<.001). In the multivariable logistic model, usage frequency was also significantly associated with delay (path *b*: OR 1.40, 95% CI 1.28-1.55; *P*<.001). The product term (indirect effect *a* × *b*) was calculated to be 0.1949, and the Sobel test yielded a significant result (*P*<.001), indicating a statistically robust mediation path (Table 3). To further validate these findings, a nonparametric bootstrap analysis with 500 replications was conducted. The mean indirect effect was found to be 0.2152 (OR 1.24, 95% CI 1.20-1.29), with a bootstrap *P* value of <.001. This confirms that AI trust contributes to delay behavior partly through increased frequency of AI usage. The direct effect of AI trust on delay remained statistically significant after adjusting for the mediator (path *c’*: OR 1.09, 95% CI 1.00-1.18; *P*=.04), supporting the conclusion of a partial mediation model (Table 3). A diagram illustrating this mediation relationship can be found in Figure 3.

**Table 3:** Mediation effect of artificial intelligence (AI) trust on health care delay behavior.

Path	β (log OR^a^)	OR (95% CI)	SE	*P* value	*P* value (Sobel)
*a* (AI trust-AI frequency)	.5754	N/A^b^	0.014	<.001	N/A
*b* (AI frequency-delay)	.3387	1.4031 (1.28-1.53)	0.0455	<.001	N/A
*a* × *b* (indirect)	.1949	N/A	0.0266	N/A	<.001
*c* (AI trust-delay, total effect)	.2473	1.2806 (1.20-1.37)	N/A	N/A	N/A
*c’* (AI trust-delay, direct effect)	.085	1.0887 (1.0-1.18)	0.0423	.04	N/A

^a^OR: odds ratio.

^b^N/A: not applicable.

### No Significant Moderation by Recommendation Exposure Level

**Figure 4:**
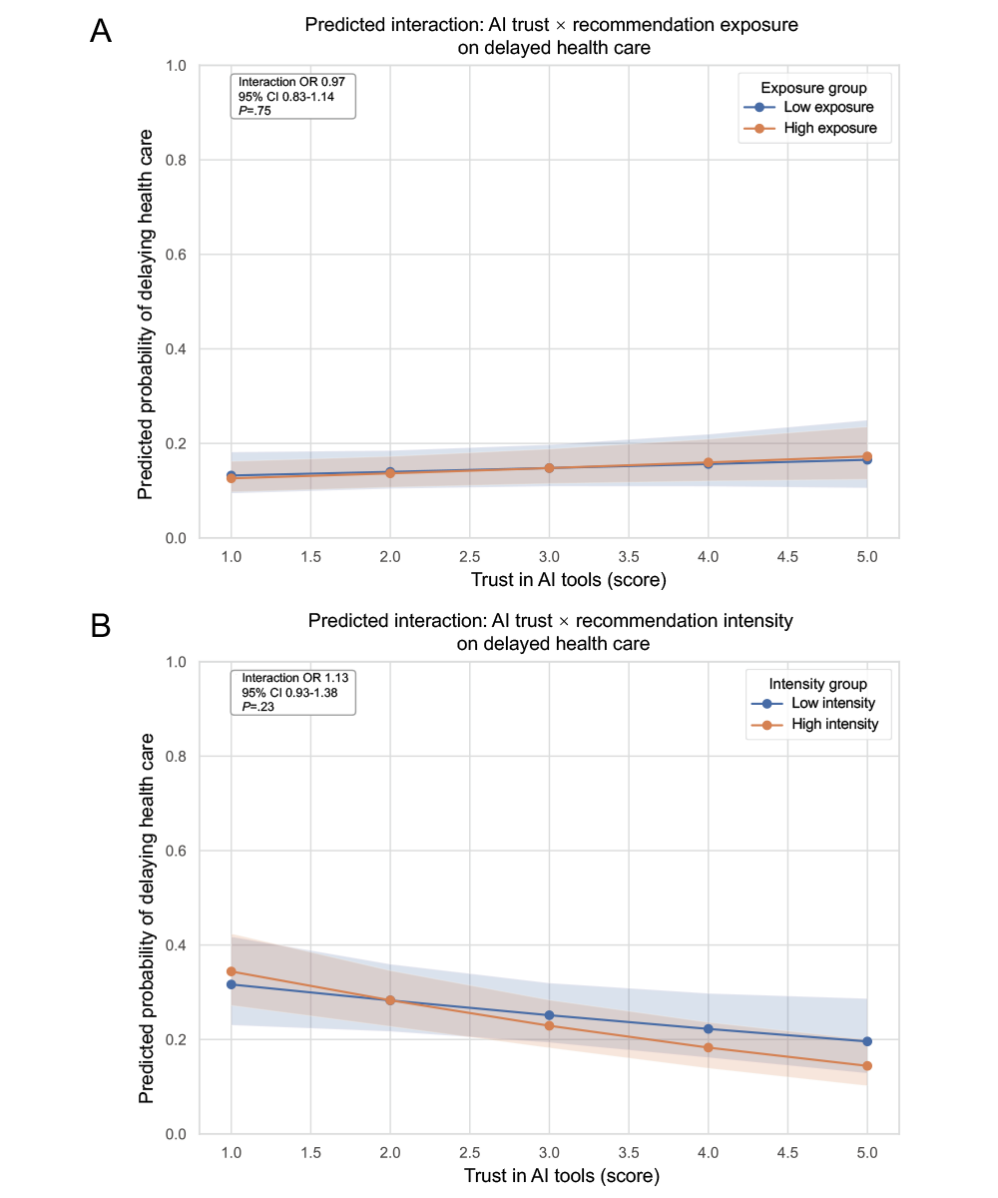
Stratified logistic regression and interaction modeling between artificial intelligence (AI) trust and recommendation exposure. Moderating effect of (A) AI recommendation exposure and (B) recommendation intensity on health care–seeking behavior. OR: odds ratio.

In the stratified analysis by recommendation exposure, AI trust was significantly associated with delayed health care–seeking in the high exposure group (OR 1.11, 95% CI 1.01-1.23; *P*=.03), but not in the low exposure group (OR 1.04, 95% CI 0.88-1.22; *P*=.67). Notably, frequency of AI use was consistently associated with delay across both groups (low: OR 1.47, 95% CI 1.24-1.73; *P*<.001; high: OR 1.38, 95% CI 1.24-1.53; *P*<.001), suggesting a stable effect regardless of exposure level (Table 4). In the full model with interaction terms, the AI trust × recommendation exposure interaction was not statistically significant (interaction OR 0.97, 95% CI 0.83-1.14; *P*=.75; Figure 4A; detailed stratified analyses can be found in Table S4 in Multimedia Appendix 2). As shown in the predicted probability plot, the slopes of AI trust on delay were similar between groups, with overlapping CIs, supporting the absence of a significant interaction (Figure 4A).

In summary, although AI trust showed a stronger association with health care delay in the high exposure group, the overall model did not indicate a statistically significant moderation effect. This suggests that while recommendation exposure may influence the magnitude of the trust-delay relationship, it does not alter its fundamental nature.

**Table 4:** Stratified analysis by recommendation exposure for the artificial intelligence (AI) trust-health care delay associations.

Outcome and variable			OR^a^ (95% CI)	*P* value
**Low recommendation exposure**				
	Trust in AI	1.04 (0.88-1.22)		<.001
	Frequency of AI use	1.47 (1.24-1.73)		.67
	Chronic disease (yes)	0.80 (0.47-1.37)		<.001
	Age	1.01 (0.99-1.04)		.42
	Gender (male)	1.35 (0.84-2.19)		.16
**High recommendation exposure**				
	Trust in AI	1.11 (1.01-1.23)		.21
	Frequency of AI use	1.38 (1.24-1.53)		<.001
	Chronic disease (yes)	1.78 (1.30-2.44)		.03
	Age	1.00 (0.99-1.01)		<.001
	Gender (male)	1.17 (0.87-1.58)		<.001

^a^OR: odds ratio.

### Limited Moderation by Recommendation Willingness Intensity

When stratified by willingness to recommend AI tools, AI trust was significantly associated with higher odds of health care delay in the low-intensity group (OR 1.16, 95% CI 1.00-1.34; *P*=.047), but not in the high-intensity group (OR 1.06, 95% CI 0.96-1.17; *P*=.27). Frequency of AI use remained a significant predictor of delay across both strata (low: OR 1.35, 95% CI 1.15-1.57; *P*<.001; high: OR 1.43, 95% CI 1.28-1.60; *P*<.001; Table 5). In the interaction model, the interaction term between AI trust and recommendation intensity was not statistically significant (interaction OR 1.13, 95% CI 0.93-1.38; *P*=.23; Figure 4B; detailed stratified analyses can be found in Table S5 in Multimedia Appendix 2). The interaction plot showed that the predicted probability of delaying care decreased with increasing AI trust in both groups, with overlapping CIs, indicating no significant moderation effect (Figure 4B).

In summary, while AI trust appears to be more strongly associated with the odds of health care delay in the low-intensity group, recommendation intensity did not significantly moderate this association statistically. Its influence may reflect subtle variation in effect size rather than a true interaction.

**Table 5:** Stratified analysis by recommendation intensity for the artificial intelligence (AI) trust-health care delay associations.

Outcome and variable		OR^a^ (95% CI)	*P* value
**Low recommendation intensity**			
	Trust in AI	1.16 (1.00-1.34)	.047
	Frequency of AI use	1.35 (1.15-1.57)	<.001
	Chronic disease (yes)	1.53 (0.94-2.48)	.09
	Age	1.00 (0.98-1.02)	.75
	Gender (male)	1.07 (0.68-1.68)	.79
**High recommendation intensity**			
	Trust in AI	1.06 (0.95-1.17)	.27
	Frequency of AI use	1.43 (1.28-1.60)	<.001
	Chronic disease (yes)	1.41 (1.02-1.95)	.04
	Age	1.00 (0.99-1.02)	.71
	Gender (male)	1.30 (0.96-1.77)	.09

^a^OR: odds ratio.

### Scenario Simulations Reveal Combined Risk of AI Trust, Frequency, and Chronic Disease

In the multivariable logistic regression analysis, all 3 key predictors—AI trust, frequency of use, and chronic disease status—were significantly associated with health care delay. Specifically, each unit increase in AI trust was linked to 9% higher odds of delay (OR 1.09, 95% CI 1.00-1.18; *P*=.04). Frequency of AI use demonstrated an even stronger association (OR 1.40, 95% CI 1.28-1.53; *P*<.001), and chronic disease status significantly increased the odds of delay as well (OR 1.42, 95% CI 1.09-1.86; *P*=.01; Table 6).

**Table 6:** Multivariable logistic regression analysis for the associations between key predictors and health care delay.

Variable	OR^a^ (95% CI)	*P* value
Trust in AI^b^	1.09 (1.00-1.18)	.04
Frequency of AI use	1.40 (1.28-1.53)	<.001
Chronic disease (yes)	1.42 (1.08-1.86)	.01

^a^OR: odds ratio.

^b^AI: artificial intelligence.

Scenario simulations indicated that, compared to a baseline of moderate trust and usage without a chronic condition (predicted probability=11.6%), increasing either AI trust (13.97%) or frequency (25.2%) alone raised the likelihood of delayed health care. The effect was most pronounced with frequency increases. When chronic illness was present alone, the predicted delay probability rose to 14.1%. Combining high trust and high frequency led to a delay probability of 30.5%, and when all 3 factors—high trust, high frequency, and chronic illness—were present, the probability escalated to 35.8%. These findings suggest that while increased trust and usage may enhance engagement with AI, they may paradoxically elevate the odds of behavioral delay due to potential overreliance or false reassurance. Therefore, intervention strategies should carefully balance cognitive trust with medical decision accuracy to mitigate potential adverse outcomes.

### Agent-Based Simulation Reveals Bidirectional Feedback Between Trust and Delay

**Figure 5:**
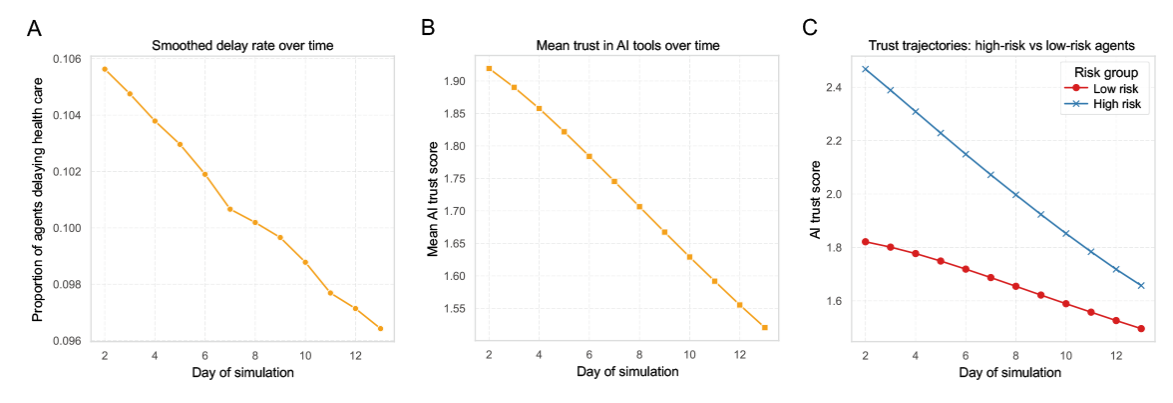
Agent-based modeling for the bidirectional feedback between artificial intelligence (AI) trust and delay behavior over a 14-day simulation period. (A) The overall rate of delayed health care. (B) The mean AI trust score. (C) AI trust score for the high-risk and low-risk groups.

Simulation results demonstrated a consistent decline in the overall rate of health care delay, decreasing from 10.6% on day 1 to 9.5% by day 14 (Figure 5A). This trend reflects behavioral feedback, where agents who experienced delays reduced their AI usage frequency, ultimately lowering delay probabilities at the population level. Concurrently, the mean AI trust score declined steadily from approximately 1.95 to 1.49 over the 14-day period (Figure 5B). This indicates a natural erosion of trust in the absence of external reinforcement, suggesting a feedback loop where behavioral delays contribute to progressive trust deterioration. When stratifying agents by their predicted baseline delay probability, both high-risk and low-risk groups exhibited declining trust; however, the high-risk group experienced a steeper decline (from 2.55 to 1.74) compared to the low-risk group (from 1.84 to 1.53; Figure 5C). This suggests that high-risk individuals may fall into a “high expectation-high disappointment” loop, accelerating trust collapse and reinforcing delay behavior. Detailed analyses for ABM can be found in Table S6 in Multimedia Appendix 2.

Overall, these simulations demonstrate the reciprocal feedback between trust and behavior, with individual heterogeneity compounding over time. The findings highlight the potential need for risk-stratified interventions or strategies designed to counteract trust erosion to prevent systemic trust breakdown.

### Strategy Comparison: Broadcast Is the Most Effective, While Rewire May Backfire

**Figure 6:**
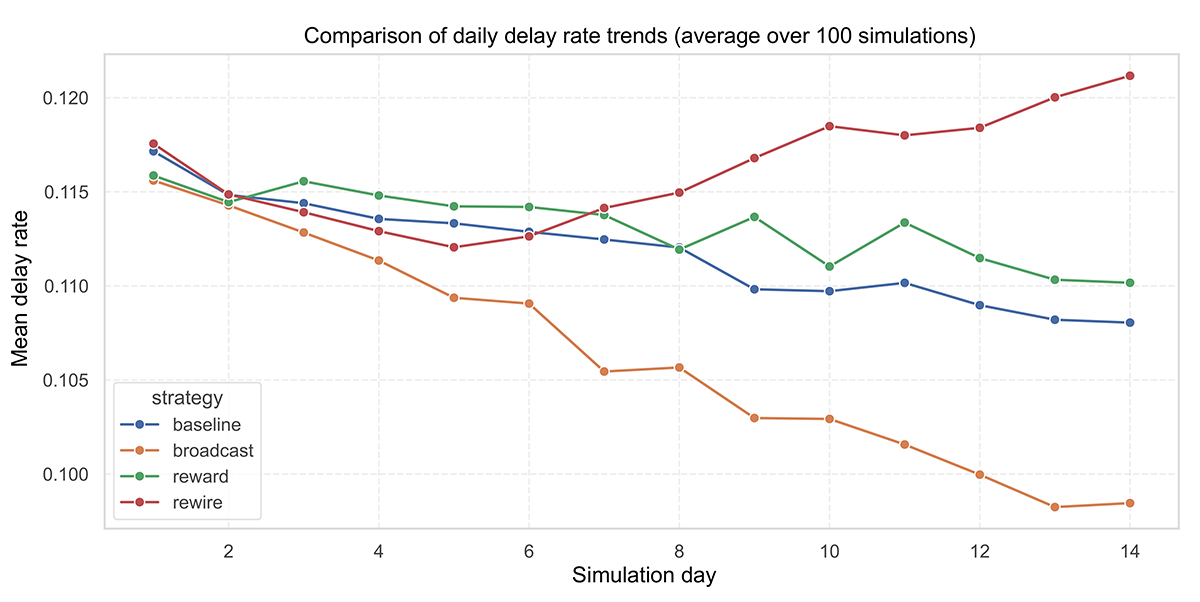
Comparison of 4 intervention strategies on delay behavior using artificial intelligence simulations. Strategies include baseline, broadcast messaging, behavioral reward, and network rewiring.

Across 100 simulation trials, we examined the temporal effects of 4 intervention strategies on delay behavior. The broadcast strategy resulted in the most significant reduction in delay rates, reaching approximately 9.7% by day 14 (Figure 6). The reward strategy showed a moderate effect, with a slight downward trend compared to the baseline (Figure 6). In contrast, the rewire strategy led to an upward trend in delay rates after day 7, ultimately surpassing the baseline group, which suggests a potential amplification of trust polarization (Figure 6).

Logistic regression further quantified these effects (Table 7). Compared to the baseline, the broadcast strategy significantly reduced the odds of delay (OR 0.94, 95% CI 0.94-0.95; *P*<.001). The reward strategy indicated a slight increase in the odds of delay (OR 1.01, 95% CI 1.01-1.02; *P*<.001), while the rewire strategy demonstrated the most substantial increase in odds (OR 1.04, 95% CI 1.04-1.05; *P*<.001), indicating that this approach may exacerbate delay behavior under certain conditions. In summary, the broadcast strategy emerged as the most effective in reducing delay behavior within the current simulation framework, highlighting the potential advantages of proactive information signaling and group-level risk awareness alerts.

**Table 7:** Logistic regression analysis for the intervention strategies on health care delay.

Strategy	OR^a^ (95% CI)	*P* value
Broadcast	0.94 (0.94-0.95)	<.001
Reward	1.01 (1.01-1.02)	<.001
Rewire	1.04 (1.04-1.05)	<.001

^a^OR: odds ratio.

To validate the robustness of the simulation across varying intervention configurations, we performed sensitivity analyses on 4 key parameters: initial trust level, broadcast penalty intensity, reward magnitude, and rewiring frequency. When varying the initial trust level (2.5, 3.0, and 3.5), higher values led to significantly increased rates of health care delay over time, suggesting that excessive trust may backfire despite its general benefits (Figure S1A in Multimedia Appendix 3). For broadcast penalty intensity, tested at values of 0.05, 0.1, and 0.2, the delay trajectories remained nearly identical, indicating minimal impact of penalty strength on intervention stability (Figure S1B in Multimedia Appendix 3). Similarly, altering the reward magnitude (0.03, 0.05, and 0.10) produced overlapping delay curves, demonstrating the strategy’s robustness and absence of nonlinear effects (Figure S1C in Multimedia Appendix 3). In the case of rewiring frequency (2, 5, and 10 days), more frequent rewiring only slightly reduced early-stage delays, with long-term outcomes remaining consistent across intervals (Figure S1D in Multimedia Appendix 3). Overall, these results confirm that the simulation framework is stable, with behavioral outcomes staying similar across a range of parameters, highlighting the broader relevance and effectiveness of the modeled interventions.

## Discussion

### Principal Findings

This study is the first to systematically investigate the joint influence of AI trust, AI usage frequency, and chronic disease status on predicting delayed health care–seeking from the perspective of a behavior-trust feedback mechanism in China. Our findings demonstrate that both AI trust and usage frequency are significant predictors of health care delay. Importantly, AI trust not only indirectly elevates the odds of delay through increased usage frequency but also exerts a significant direct association when controlling for usage frequency, indicating a partial mediation pathway. Of note, individuals with chronic conditions inherently exhibit a predisposition toward delayed care. These odds are further exacerbated by the combined effects of high AI trust and frequent AI use. These results expand the current understanding of delayed health care behaviors. They emphasize the complex and dynamic interplay between technological trust and health-related decision-making. Additionally, they highlight that enhanced trust in AI may inadvertently contribute to adverse behavioral outcomes among patients with chronic diseases.

To investigate the psychological mechanisms underlying the “AI trust-frequent AI use-health care delay” pathway, we propose that cognitive bias and overreliance are key driving factors [31,32]. On one hand, patients with chronic conditions often experience stable and slowly progressing symptoms. They are more likely to view AI’s reassuring suggestions as indicators of safety. As a result, they may underestimate the seriousness of their own symptoms. This sense of “false reassurance” is particularly pronounced among individuals with high levels of trust in AI [33]. Previous studies have shown that excessive trust in health AI recommendations can result in users neglecting bodily warning signs, ultimately leading to delays in health care–seeking [5]. On the other hand, frequent AI usage does not necessarily reflect higher health literacy. It also does not always indicate better self-management capacity [34]. Instead, it may suggest a psychological tendency to avoid traditional health care services. This tendency is especially common when medical care is expensive, time-consuming, or perceived as untrustworthy [35]. This form of “instrumental dependence” may drive individuals to rely on AI as a substitute source of advice when experiencing discomfort, instead of seeking timely professional care [36]. Therefore, this study emphasizes that the behavioral consequences of AI trust and usage frequency should not be interpreted simply as empowerment. Instead, it is important to explore the underlying psychological and behavioral mechanisms. This is essential for accurately assessing the true impact of technological interventions on health care behavior.

Although we further investigated the moderating effects of AI recommendation exposure and recommendation willingness on the “AI trust-health care delay” pathway, the interaction terms were not statistically significant. This indicates that these factors have a limited influence on the primary effect pathway. Subgroup analyses revealed some trend-level differences; for instance, individuals with higher recommendation willingness showed a slight reduction in delay behavior [37], but these effects did not achieve statistical significance. We speculate that a nonlinear threshold effect may be present. Once recommendation exposure reaches a certain frequency, its impact may plateau. This could result in a failure to further enhance trust or promote behavioral change. Another possibility is that the effectiveness of recommendations is highly context dependent. They may exert influence primarily when users experience high health anxiety, evident symptoms, or a strong sense of urgency [38,39]. Additionally, AI trust may be inherently unstable among individuals, easily influenced by emotional states, prior experiences, or public opinion [40]. These factors may further diminish the practical effectiveness of recommendations. While recommendation behaviors have the potential for positive influence, their underlying mechanisms appear more complex [41]. They may not adequately address the fundamental contradiction between AI trust and health care delay. Therefore, future interventions should not concentrate solely on increasing recommendation frequency or willingness. Instead, it is crucial to address system-level design issues. This includes enhancing decision transparency and improving users’ perceived control over health-related choices. Additionally, developing prompting strategies that better align with users’ psychological models is essential. Such improvements are essential for facilitating meaningful and lasting behavioral change.

Scenario-based simulations further confirmed that the combination of high AI trust, frequent AI usage, and chronic disease status results in the greatest odds of delayed health care–seeking. This finding underscores the behavioral risks inherent in the trust feedback mechanism. Using behavioral decision theories, particularly the dual-process model, helps illuminate the psychological underpinnings of this phenomenon [42]. In high-trust situations, individuals tend to rely more on the intuitive system for decision-making, leading to quick, automatic judgments rather than thorough risk evaluations [43]. This “intuitive trust” is especially evident among patients with chronic illnesses, who often feel a sense of safety due to the relatively stable nature of their symptoms. When coupled with high trust in and frequent reliance on AI, these individuals are more likely to accept AI-generated suggestions without critically assessing their health status or seeking professional medical advice [44]. This can create a “false reassurance” effect, significantly heightening the risk of delayed health care. These findings serve as a cautionary note for the future design of AI-based health tools. Simply pursuing user trust is insufficient; it is essential to strike a balance between fostering trust and maintaining risk awareness. Both clinical practitioners and AI developers need to consider how to enhance user experience while mitigating the unintended consequences of overreliance [45]. Future AI systems should be designed to promote rational judgment, especially among high-risk groups with chronic conditions. Incorporating interactive features that provide calibrated risk reminders may be necessary to prevent the unconscious shift from trust to delayed health care behavior.

Our ABM offered a dynamic and systems-level understanding of how AI trust and delay behavior coevolve over time. Unlike traditional regression models that provide static snapshots [23], ABM captures how small differences in initial trust and exposure can compound through individual learning and social influence. Over a 14-day simulation period, we observed a steady decline in both trust and health care–seeking behavior, indicating a reciprocal erosion loop: delayed health care leads to dissatisfaction or unmet expectations, which in turn diminishes trust and further health care delay [46]. Importantly, agents stratified by predicted baseline odds (above vs below 20%) showed divergent trust trajectories. High-risk individuals began with higher trust but experienced a sharper decline—suggesting a “high expectation-high disappointment” loop. This highlights how perceived safety in chronic illness, when coupled with excessive AI reliance, may paradoxically lead to trust collapse and amplified delay behavior. Such patterns would be difficult to detect using empirical data alone, underlining the value of simulation modeling in behavioral health research. Beyond reproducing observed behaviors, ABM also allowed us to simulate system-wide interventions and uncover nonlinear responses to trust regulation strategies. Of the 3 mechanisms tested, broadcast messaging consistently reduced delay by maintaining population-level risk awareness. In contrast, network rewiring unexpectedly increased delay, likely due to the formation of echo chambers among high-trust individuals, a phenomenon we term “trust polarization” [47]. This emergent property demonstrates that even well-intentioned peer-based reinforcement may backfire under certain trust dynamics. Overall, these simulations underscore the importance of viewing AI trust not merely as an individual attribute, but as a collective behavioral variable that evolves across time, context, and networks [48]. While the ABM provides valuable insights into potential system dynamics and the comparative theoretical performance of different strategies, these findings serve as a proof of concept. Their real-world effectiveness and causal impact must be rigorously tested in future randomized controlled trials or natural experiments.

Simulation-based evaluations of 3 intervention strategies indicate that broadcast messaging is the most effective for reducing delayed health care behavior. This suggests that public health interventions delivering wide-reaching, consistent risk reminders may be the most cost-effective approach [49]. Conversely, the reward-based strategy was less effective, likely due to insufficient incentives or limited reach, which hindered meaningful behavior change at the population level [50]. Unexpectedly, the network rewiring strategy not only failed to reduce delays but exacerbated them. We hypothesize that this may result from the formation of information echo chambers among high-trust individuals [51]. In these tightly connected groups, AI trust can become mutually reinforced, amplifying the “AI trust-health care delay” pathway and leading to what we term trust polarization. These findings underscore the need to prioritize intervention strategies that broadly disseminate risk information and continuously enhance risk awareness. Future health interventions should focus on scalable and sustainable communication mechanisms rather than relying on individual “opinion leaders” or short-term incentives. This approach provides valuable insights into integrating AI and health behavior, while also offering empirical evidence to inform public health policy development.

### Limitations

Despite proposing a novel “trust-behavior” feedback mechanism and validating various intervention strategies through simulation, this study has several limitations. First, the cross-sectional and observational nature of our survey data precludes definitive causal inference. While we controlled for several demographic and health factors, the potential for omitted variable bias remains. Unmeasured confounders, such as general health literacy and prior negative experiences with the health care system, could independently influence both AI trust and the propensity to delay care. To establish causality, future research should use randomized controlled trials that investigate participants’ trust in AI. This approach would enable more conclusive mediation and moderation analyses and would represent a critical continuation of our future research efforts. Second, measurements of AI trust and usage frequency were based on self-reported data. This approach may introduce recall bias, social desirability effects, or subjective judgment. Consequently, these factors could potentially affect the accuracy of the findings. Although we introduced a group-based behavioral simulation model to address the static nature of cross-sectional data, this model simplifies the complex dynamics of trust evolution in real-world contexts. Future research could incorporate reinforcement learning or dynamic trust modeling approaches to better capture human cognitive and behavioral trajectories [52]. Third, the generalizability of our findings may be limited by the specific sociotechnical context of China. Our recruitment relied on dominant Chinese digital platforms (WeChat and QQ). The observed relationships between AI trust, usage, and health care delays are likely influenced by China’s unique health care system, technology ecosystem, and cultural norms. Therefore, caution is warranted when extending these results to other countries with different health care policies, technology adoption patterns, and cultural attitudes toward AI in medicine. Fourth, our recruitment via social media and hospital portals, while enabling broad access, prevents precise quantification of response rates from each channel. Although we estimate that most responses came from WeChat and QQ, with a smaller proportion from hospital portals, the anonymous nature of the survey precluded channel-specific stratification. Additionally, this approach likely oversampled individuals who are more digitally literate or proactively engaged with health care information. This may limit the generalizability of our findings to populations with limited digital access or lower health literacy. Fifth, the questionnaire did not collect data on rural or urban residence or socioeconomic status, limiting our ability to examine these potential sociodemographic influences on the trust-delay pathway. Future studies would benefit from incorporating these variables. Sixth, our sample contained a slightly higher proportion of female participants (1345/2460, 54.7%), which may influence generalizability. However, we adjusted for gender in all analyses and found no evidence of significant effect modification. Finally, we advocate for the development of AI health tools with stratified trust management functions [53]. Such systems should provide tailored risk alerts for high-risk patients with chronic conditions, promoting rational trust for genuine health empowerment rather than fostering passive dependence.

### Conclusions

This study identifies a nuanced relationship between AI trust and delayed health care–seeking behavior. While trust in AI tools can enhance user engagement, it may also be associated with delayed health care, particularly among individuals with chronic conditions or higher levels of AI use. By integrating survey data with ABM, our study moves beyond static associations and illustrates how AI trust and delay behaviors may interact and reinforce one another over time. This dynamic perspective highlights how individual-level trust processes can accumulate into system-level patterns, including trust polarization and collective delay. Our findings suggest that the design of AI health tools should prioritize calibrated decision support rather than full automation. Encouraging a balanced interaction between user autonomy and technological assistance may help mitigate delay-related risks and improve decision quality in digital health contexts. Beyond individual-level tool design, these insights may also inform population-level strategies for trust governance and risk communication in AI-driven health care systems.

## Appendix

Multimedia Appendix 1 English-translated survey questionnaire.

Multimedia Appendix 2 Supplementary Tables S1-S6.

Multimedia Appendix 3 Supplementary Figure S1.

## Abbreviations

ABM: agent-based modeling
AI: artificial intelligence
MCAR: missing completely at random
ODD: overview, design concepts, and details
OR: odds ratio

## Data Availability

The datasets generated or analyzed during this study are available from the corresponding author on reasonable request.
