# Supplementary material for: Behavioral Dynamics of AI Trust and Health Care Delays Among Adults: Integrated Cross-Sectional Survey and Agent-Based Modeling Study
Source: J Med Internet Res. 2026 Feb 3;28:e82170. doi: 10.2196/82170 (PMC12914233; doi:10.2196/82170)
Supplement: Multimedia Appendix 1 [file jmir_v28i1e82170_app1.docx]

1. **What is your gender?**
   ☐ Male
   ☐ Female
2. **What is your age?** (Please fill in an integer between 18–75)
3. **What is your occupation?**
   ☐ Student (including medical students)
   ☐ Technology professional
   ☐ Healthcare worker
   ☐ Civil servant/Teacher
   ☐ Finance/Legal related
   ☐ Retired
   ☐ Other: ________
4. **Have you heard of ChatGPT or similar AI tools?**
   ☐ Yes
   ☐ No
5. **Have you ever used ChatGPT or similar AI tools for health consultation?**
   ☐ Yes
   ☐ No
6. **If you have used it, how often do you use it?**
   ☐ 0 = Never
   ☐ 1 = Occasionally
   ☐ 2 = A few times a month
   ☐ 3 = 1–2 times a week
   ☐ 4 = 3–4 times a week
   ☐ 5 = Almost daily
7. **How much do you trust the health advice provided by AI? (1–5 points)**
   ☐ 1 = Not trusting at all
   ☐ 5 = Very trusting
8. **Has the advice from ChatGPT influenced you to change your original medical decision?**
   ☐ Yes
   ☐ No
9. **Has the advice from ChatGPT caused you to delay or cancel medical appointments?**
   ☐ Yes
   ☐ No
10. **Has the advice from ChatGPT prompted you to take alternative treatments different from traditional ones?**
    ☐ Yes
    ☐ No
11. **What is your main source of health information?**
    ☐ Doctor
    ☐ Baidu / Google
    ☐ Family
    ☐ Friends
    ☐ Policy promotion
    ☐ AI tools (e.g., ChatGPT)
12. **Does community discussion affect your health decisions?**
    ☐ Definitely not
    ☐ Probably not
    ☐ Uncertain
    ☐ Probably
    ☐ Definitely
13. **Do you habitually discuss health issues publicly on social platforms?**
    ☐ Yes
    ☐ No
14. **Does policy or public health advocacy affect your level of trust in AI? (1–5 points)**
15. **Do you have any chronic diseases?**
    ☐ Yes
    ☐ No
16. **Does policy promotion enhance your trust in tools like GPT? (1–5 points)**
17. **Does a doctor's recommendation enhance your trust in tools like GPT? (1–5 points)**
18. **Does the technological aspect of GPT make you more apprehensive about medical decisions? (1–5 points)**
19. **Have you avoided seeking medical care due to GPT's suggestions? (1–5 points)**
20. **Have you proactively recommended GPT tools for health purposes to others? (1–5 points)**
21. **Have you ever been recommended by others to use GPT tools? (1–5 points)**
