# Supplementary material for: Behavioral Dynamics of AI Trust and Health Care Delays Among Adults: Integrated Cross-Sectional Survey and Agent-Based Modeling Study
Source: J Med Internet Res. 2026 Feb 3;28:e82170. doi: 10.2196/82170 (PMC12914233; doi:10.2196/82170)
Supplement: Multimedia Appendix 2 [file jmir_v28i1e82170_app2.docx]

**Supplementary Table S1.** Univariate logistic regression to evaluate predictors of healthcare seeking delays

| Variable | OR | 95% CI | *P* value |
| --- | --- | --- | --- |
| AI trust | 1.27 | 1.19–1.36 | < 0.001 |
| Age | 1.00 | 0.99–1.01 | 0.6802 |
| Gender (Male) | 1.19 | 0.93–1.52 | 0.1712 |
| Chronic disease (Yes) | 1.30 | 1.00–1.68 | 0.0505 |
| Policy trust boost | 1.07 | 0.95–1.21 | 0.2524 |
| Doctor’s recommendation boost | 1.04 | 0.93–1.15 | 0.5095 |
| Scientific deterrent effect | 0.91 | 0.82–1.01 | 0.0711 |
| Frequency of AI use | 1.46 | 1.35–1.57 | < 0.001 |
| Receiving AI recommendation | 0.95 | 0.86–1.05 | 0.3212 |
| Actively recommended AI | 0.99 | 0.89–1.10 | 0.8443 |

OR, odds ratio; CI, confidence interval

**Supplementary Table S2.** Multivariate logistic regression (Model 4) to evaluate predictors of healthcare seeking delays

| Variable | OR | 95% CI | *P* value |
| --- | --- | --- | --- |
| AI trust | 0.04 | 0.02–0.11 | 0.043 |
| Age | 1.09 | 1.00–1.18 | 0.7069 |
| Gender (Male) | 1.00 | 0.99–1.01 | 0.1145 |
| Chronic disease (Yes) | 1.23 | 0.95–1.58 | 0.0087 |
| Policy trust boost | 1.43 | 1.10–1.88 | 0.2482 |
| Doctor’s recommendation boost | 1.08 | 0.95–1.22 | 0.7244 |
| Scientific deterrent effect | 1.02 | 0.91–1.14 | 0.0788 |
| Frequency of AI use | 0.91 | 0.82–1.01 | < 0.001 |
| Receiving AI recommendation | 1.40 | 1.28–1.53 | 0.4126 |
| Actively recommended AI | 0.96 | 0.86–1.06 | 0.8421 |

OR, odds ratio; CI, confidence interval

**Supplementary Table S3.** Univariate logistic regression to evaluate predictors of healthcare seeking delays before and after DeepSeek release

| Periods | Variable | OR | 95% CI | *P* value |
| --- | --- | --- | --- | --- |
| Before DeepSeek | AI trust | 1.05 | 0.89–1.25 | 0.561 |
|  | Age | 1.03 | 1.00–1.05 | 0.0236 |
|  | Gender (Male) | 1.08 | 0.63–1.84 | 0.7848 |
|  | Chronic disease (Yes) | 1.37 | 0.78–2.40 | 0.2735 |
|  | Frequency of AI use | 1.41 | 1.17–1.70 | 0.0003 |
| After DeepSeek | AI trust | 1.10 | 1.00–1.21 | 0.0464 |
|  | Age | 1.00 | 0.98–1.01 | 0.5547 |
|  | Gender (Male) | 1.25 | 0.94–1.67 | 0.1291 |
|  | Chronic disease (Yes) | 1.46 | 1.07–1.98 | 0.0156 |
|  | Frequency of AI use | 1.41 | 1.27–1.56 | < 0.001 |

OR, odds ratio; CI, confidence interval

**Supplementary Table S4.** Analysis of interaction term between AI trust and recommendation exposure

| Variable | Coef (log-OR) | OR | 95% CI | *P* value |
| --- | --- | --- | --- | --- |
| Low recommendation exposure group | 0.0795 | 1.08 | 0.67–1.76 | 0.7473 |
| AI trust | 0.0921 | 1.10 | 1.00–1.20 | 0.0528 |
| AI trust × Low recommendation | -0.0262 | 0.97 | 0.83–1.14 | 0.7497 |
| Frequency of AI use | 0.3392 | 1.40 | 1.28–1.53 | < 0.001 |
| Chronic disease (Yes) | 0.3623 | 1.44 | 1.10–1.88 | 0.0082 |
| Age | 0.0028 | 1.00 | 0.99–1.01 | 0.6134 |
| Gender (Male) | 0.2029 | 1.23 | 0.95–1.58 | 0.1174 |

OR, odds ratio; CI, confidence interval

**Supplementary Table S5.** Analysis of interaction term between AI trust and recommendation exposure

| Variable | Coef (log-OR) | OR | 95% CI | *P* value |
| --- | --- | --- | --- | --- |
| Low recommendation intensity group | -0.2478 | 0.78 | 0.42–1.44 | 0.4291 |
| AI trust | -0.284 | 0.75 | 0.67–0.84 | < 0.001 |
| AI trust × Low recommendation | 0.1234 | 1.13 | 0.93–1.38 | 0.2271 |
| Frequency of AI use | -0.0902 | 0.91 | 0.82–1.02 | 0.0966 |
| Chronic disease (Yes) | 0.3462 | 1.41 | 1.07–1.87 | 0.0146 |
| Age | 0.0017 | 1.00 | 0.99–1.01 | 0.7697 |
| Gender (Male) | 0.1897 | 1.21 | 0.93–1.57 | 0.156 |

OR, odds ratio; CI, confidence interval

**Supplementary Table S6.** Agent-based modeling for the rate of healthcare delay

| Day | Delay rate (%) | Mean trust score | Trust score (high-risk) Trust score (low-risk) | |
| --- | --- | --- | --- | --- |
| 1 | 0.106223577 | 1.946104065 | 1.838924772 | 2.545790885 |
| 2 | 0.104703252 | 1.920308537 | 1.829253136 | 2.499831257 |
| 3 | 0.103760163 | 1.891369919 | 1.817098901 | 2.44870065 |
| 4 | 0.103971545 | 1.858630081 | 1.801825965 | 2.39294087 |
| 5 | 0.102678862 | 1.822310569 | 1.783088765 | 2.333336313 |
| 6 | 0.102036585 | 1.783504878 | 1.761079744 | 2.27032912 |
| 7 | 0.101207317 | 1.745732114 | 1.737021268 | 2.20536357 |
| 8 | 0.099857724 | 1.70635813 | 1.710746383 | 2.13889824 |
| 9 | 0.099219512 | 1.666038211 | 1.682410344 | 2.071419427 |
| 10 | 0.098995935 | 1.628226829 | 1.653364812 | 2.003517909 |
| 11 | 0.098284553 | 1.591086179 | 1.623726289 | 1.936478503 |
| 12 | 0.096796748 | 1.554015447 | 1.593557867 | 1.870271103 |
| 13 | 0.095195122 | 1.517578862 | 1.563133244 | 1.804876817 |
| 14 | 0.095056911 | 1.485510163 | 1.533493686 | 1.743066949 |
