# Supplementary material for: Behavioral Dynamics of AI Trust and Health Care Delays Among Adults: Integrated Cross-Sectional Survey and Agent-Based Modeling Study
Source: J Med Internet Res. 2026 Feb 3;28:e82170. doi: 10.2196/82170 (PMC12914233; doi:10.2196/82170)
Supplement: Multimedia Appendix 3 [file jmir_v28i1e82170_app3.docx]

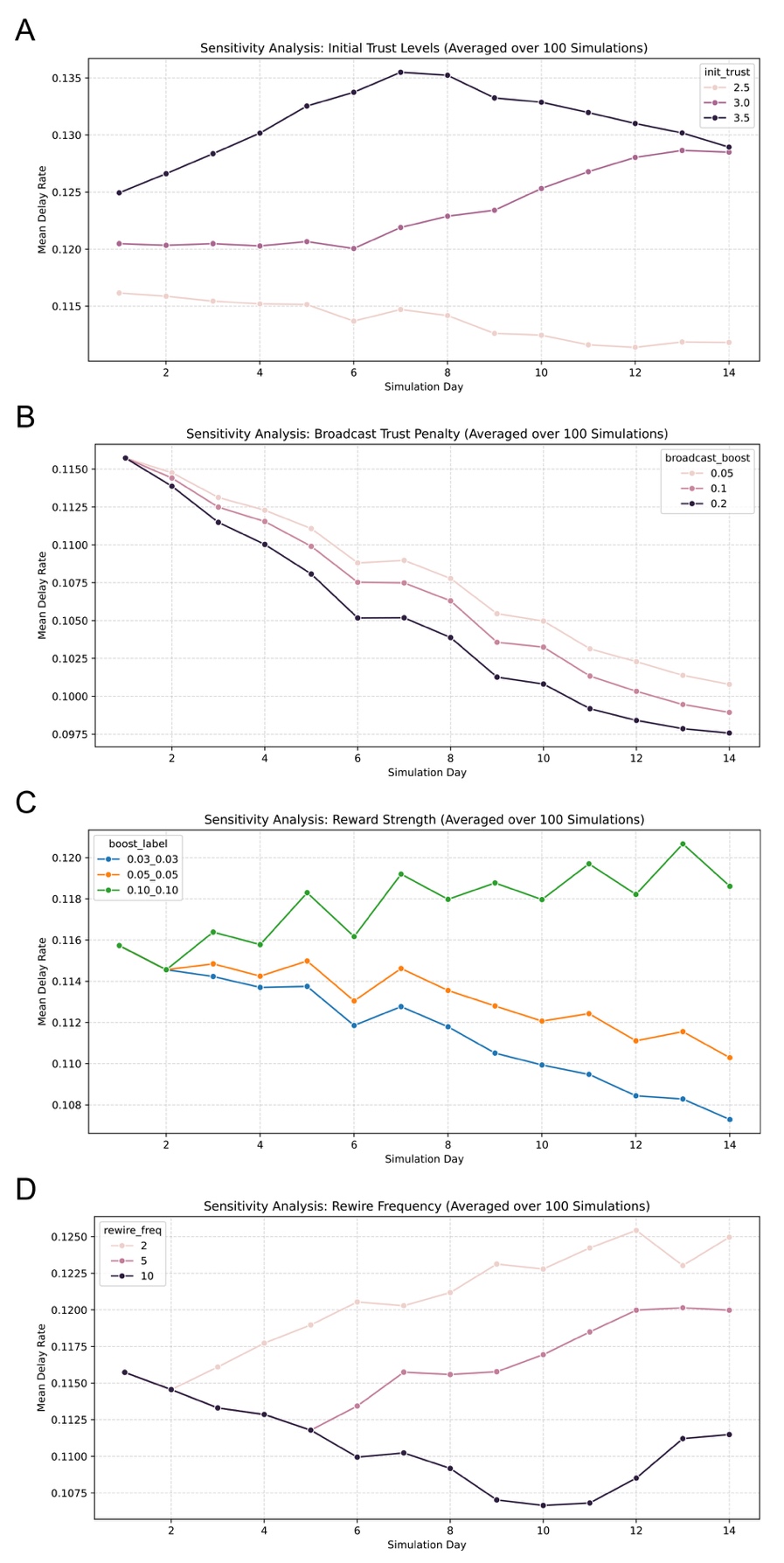


**Supplementary Figure S1.** Sensitivity analysis of key parameters in the agent-based modeling (ABM). The simulated population-level healthcare delay rates are presented over a 14-day period under variations of four model parameters: (A) initial AI trust level, (B) broadcast penalty intensity, (C) reward magnitude, and (D) rewiring frequency. Each panel displays the average delay rate trajectory across 100 simulation trials for different parameter values, allowing for visual comparison of how each parameter influences the model's output.
